# Epigenetic Clock Deceleration and Maternal Reproductive Efforts: Associations With Increasing Gray Matter Volume of the Precuneus

**DOI:** 10.3389/fgene.2022.803584

**Published:** 2022-03-01

**Authors:** Shota Nishitani, Ryoko Kasaba, Daiki Hiraoka, Koji Shimada, Takashi X. Fujisawa, Hidehiko Okazawa, Akemi Tomoda

**Affiliations:** ^1^ Research Center for Child Mental Development, University of Fukui, Fukui, Japan; ^2^ Division of Developmental Higher Brain Functions, United Graduate School of Child Development, Hamamatsu University School of Medicine, Osaka University, Kanazawa University, Chiba University, University of Fukui, Osaka, Japan; ^3^ Life Science Innovation Center, University of Fukui, Fukui, Japan; ^4^ Japan Society for the Promotion of Science, Tokyo, Japan; ^5^ Biomedical Imaging Research Center, University of Fukui, Fukui, Japan; ^6^ Department of Child and Adolescent Psychological Medicine, University of Fukui Hospital, Fukui, Japan

**Keywords:** DNA methyaltion, imaging epigenetics, longevity, maternal brain, voxel-based morphometry (VBM)

## Abstract

Reproductive efforts, such as pregnancy, delivery, and interaction with children, make maternal brains optimized for child-rearing. However, extensive studies in non-human species revealed a tradeoff between reproductive effort and life expectancy. In humans, large demographic studies have shown that this is the case for the most part; however, molecular marker studies regarding aging remain controversial. There are no studies simultaneously evaluating the relationship between reproductive effort, aging, and brain structures. We therefore examined the associations between reproductive efforts (parity status, number of deliveries, motherhood period, and cumulative motherhood period), DNA methylation age (mAge) acceleration (based on Horvath’s multi-tissue clock and the skin & blood clock), and the regional gray matter volumes (obtained through brain magnetic resonance imaging (MRI) using voxel-based morphometry) in 51 mothers aged 27–46 years of children in early childhood. We found that increasing reproductive efforts were significantly associated with decelerated aging in mothers with one to four children, even after adjusting for the confounding effects in the multiple linear regression models. We also found that the left precuneus gray matter volume was larger as deceleration of aging occurred; increasing left precuneus gray matter volume, on the other hand, mediates the relationship between parity status and mAge deceleration. Our findings suggest that mothers of children in early childhood, who have had less than four children, may benefit from deceleration of aging mediated via structural changes in the precuneus.

## Introduction

In non-human species, the established theory (LHT: Life History Theory) is that the greater the reproductive effort, the more finite energy is expended; thus, females tradeoff life expectancy with reproductive effort (Hill and Kaplan, 1999). In humans, several large, population-based demographic studies of more than 140,000 women born between 1820 and 1920 in a preindustrial population in Utah, North America, have also shown that women who undertook more reproductive effort lived a shorter lifespan (Penn and Smith, 2007; Bolund et al., 2016). These results are largely in line with the LHT. However, according to Bolund et al. (2016), maternal life expectancy is shortened with four or more births, per the LHT; conversely, deliveries of less than four extended life expectancy (Bolund et al., 2016). Our current monogamous society no longer has an average of nine births (as was common in this previous era); for example, the number of childbirths in first-time married couples who had been married for 15–19 years in Japan was 1.94 in 2015 (The Fifteenth Japanese National Fertility Survey in 2015, 2017). A more recent large, North America-wide demographic study of more than 20,000 women was conducted (Shadyab et al., 2017), which better reflects the current average number of births. Shadyab et al. (2017) reported that the odds ratio (OR) of longevity in Caucasians was higher for mothers who gave birth more than once (two to four children; mean OR: 1.13) compared to that of those who gave birth once (OR: 0.91) or were nulliparous (OR: 1 as reference), even after adjusting for demographic characteristics, socioeconomic status, lifestyle behaviors, reproductive factors, and health-related factors (Shadyab et al., 2017). However, it was consistent with both the LHT and previous population demographics in most aspects, especially regarding having more children; the association between the number of deliveries and longevity was attenuated, and was certainly not higher in those with five or more children. Dior et al. (2013) reported a U-shaped and nonlinear, association between the number of deliveries and all-cause maternal mortality; the lowest risk was at two to three children (Dior et al., 2013). Therefore, while it may be true that having more than four children reduces life expectancy in women, the LHT may not be consistent with births of less than four children.

Hence, recent studies using molecular metrics have attempted to provide biological evidence for the effects of reproductive effort on women’s longevity. Telomere length was used as a molecular metric to reflect longevity in the initial study stages. The first study reported that mothers who had more children had shorter telomere lengths (Gray et al., 2014); by contrast, a study of the Mayan tribe in Guatemala reported that mothers who had more children had longer telomeres (Barha et al., 2016). In addition to telomere length, epigenetic age acceleration—which is obtained by calculating the deviation of DNA methylation age (mAge) from chronological age (Horvath, 2013)—has recently attracted attention, serving as a novel biomarker of aging. Using this metric, Ryan et al. showed that in 397 young, 20–22 year-old Filipino women, a higher number of pregnancies was associated with a greater mAge acceleration and shorter telomere length (Ryan et al., 2018). Kresovich *et al.* also retrospectively used this metric in more than 2,000 women aged 35–74 and living in the US, reporting that increased acceleration of aging was seen with more births (Kresovich et al., 2019). Nevertheless, the results of these studies cannot sufficiently explain why the influence of births of four or fewer children might be exceptionally non-linear. Moreover, the cause of this putative possibility might be difficult to elucidate in retrospective studies on generations that have already completed child-rearing. While the participants in the study by Ryan et al. were supposed to be rearing children, the mean age of childbearing in most developed countries is considerably higher, and was as high as 30.7 years old in Japan in 2016; therefore, while their results are applicable to younger mothers, it remains uncertain whether they can be applied to the general maternal population at present.

In addition to the phenomenon where reproductive effort affects aging and longevity, it has been affirmed that reproductive effort, including the experiences of pregnancy, delivery, and parenting, makes female brain structures more maternal and optimized for parenting (Nishitani et al., 2011; Nishitani et al., 2014; Kim, 2016). Hoekzema et al. (2017) conducted a longitudinal study that followed 25 first-time mothers from time of conception to 2 years after delivery; they found that at 10 months postpartum, the gray matter (GM) volume of the inferior and medial frontal gyrus, superior temporal sulcus, fusiform gyrus, precuneus, and hippocampus—involved in the theory-of-mind network—was reduced compared with during pregnancy (Hoekzema et al., 2017). Zhang et al. (2019) examined a similar longitudinal study and found that mothers showed changes in GM and white matter volumes, as well as the cortical thickness of several regions—including the superior and medial frontal gyrus, insula, limbic lobe, superior and middle temporal gyrus, and precentral gyrus—after 2 years of follow-up (Zhang et al., 2019). Luders et al. (2020) conducted a similar longitudinal study. They reported no decline in areas at 4–6 weeks postpartum compared with the immediate postpartum period (one to two days postpartum); however, there were increased GM volumes in certain regions, including the pre- and postcentral gyrus, thalamus, and precuneus, contrary to Hoekzema et al.’s results (Luders et al., 2020). These studies suggest that reproductive effort affects not only aging, but also elicits changes in brain structure. Contrasting with other species, humans do not reach the end of their life shortly after menopause; in fact, they live nearly twice as long as their age at menopause in this modern era. The grandmother hypothesis, explaining the existence of menopause in human life history by identifying the adaptive value of extended kin networking, has been proposed (Hawkes, 2004), as postmenopausal women have been found to have a great deal of biological and social activity (Kida et al., 2014; Hawkes and Finlay, 2018). This is unique to humans and is considered to be related to humans’ high sociability, owing to their highly evolved brains (Hawkes, 2020).

Therefore, we hypothesized that reproductive effort would refine the brain structures involved in the sociability required for parenting, thereby influencing both mAge acceleration and longevity. In the present study, we examined the association between reproductive effort, mAge acceleration measured using salivary DNA, and brain structures evaluated by voxel-based morphometry (VBM), in mothers.

## Materials and Methods

### Participants

A total of 57 Japanese biological mothers rearing at least one child of preschool age participated in this study; this dataset is used for our previous studies (Hiraoka et al., 2020; Sakakibara et al., 2021; Kasaba et al., 2021; Shimada et al., 2019). We excluded four mothers who delivered by caesarean section for multiple births, and two mothers of children with disabilities. A total of 51 mothers were included (age range = 27–46 years; mean age = 34.8 years; standard deviation (SD) = 4.5 years). Detailed demographics are shown in Table 1. Among the participants, 11 had one child, 30 had two children, nine had three children, and one had four children. We defined “parity status” as primiparity or multiparity, and “number of deliveries” as the number of times a mother gave live birth; these represented the reproductive effort indices. The average age of the oldest children of the participants was 6.2 years (SD = 3.2); this age was defined as the total “motherhood period” until the day of the experiment. We added up the age of all children to calculate an index, referred to as the “cumulative motherhood period”; this reflected the efforts invested in parenting more appropriately for mothers rearing multiple children (Supplementary Figure S1 and Supplementary Table S1). All participants had received at least 12 years of education. Almost all (92.3 %) were right-handed, according to the FLANDERS handedness inventory (Nicholls et al., 2013; Okubo et al., 2014).

**TABLE 1:** Demographic characteristics of the participants.

	(*n* = 51)
Age (years), *Mean* (*SD*)	35.4 (4.4)
Age (years) at first childbirth	29.1 (4.0)
Age (years) at last childbirth	32.3 (3.9)
Parity status, *n* (%)	11 (21.6)/40 (78.4)
Primiparity/Multiparity	
Number of deliveries, *n* (%)	11 (21.6)
One	30 (58.8)
Two	9 (17.6)
Three	1 (2.0)
Four	
Motherhood period (age of oldest child) (years)	6.2 (3.2)
Cumulative motherhood period (years)	9.9 (6.0)
Exclusive breastfeeding diet, *n* (%)	30 (58.8)
Household Income (currency = JPY), *n* (%)	
Less than 3 million	3 (5.9)
3–5 million	25 (49.0)
5–10 million	21 (41.2)
More than 10 million	2 (3.9)
MRI scanner, *n* (%)	
Discovery MR 750 3T/Signa PET/MR 3T	30 (58.8)/21 (41.2)
Proportion of Epithelial cells (%)	15.9 (5.8)
FLANDERS handedness inventory (right/mixed/left)	48 (92.3)/1 (1.9)/2 (3.8)
PSI (Total/Child/Adult)	191.5 (39.8)/86.5 (18.6)/105.1 (24.6)
BDI-II	12.0 (9.2)

PSI, Parenting Stress Index (Abidin, 1995; Namara et al., 1999); BDI-II, The Beck Depression Inventory-II (Beck, Steer, & Brown, 1996; Kojima et al., 2002).

This study’s protocol was approved by the Ethics Committee of the University of Fukui (FU20150109 and FU20190107), and was conducted following the Declaration of Helsinki. All participants provided written informed consent for participation in this study.

### Questionnaires

The Japanese version of the Parenting Stress Index (PSI) (Namara et al., 1999)—an adaptation of the PSI (Abidin, 1995)—was used to evaluate the participants’ parenting stress (PSI total). The Beck Depression Inventory-II (BDI-II) (Beck et al., 1996; Kojima et al., 2002) was used to measure the participants’ depressive symptoms.

### DNA Methylation

Saliva samples were collected using Oragene Discover OGR-500 kits (DNA Genotek Inc., Ottawa, ON, Canada). Saliva DNA was extracted using prepIT^®^•L2P reagent (DNA Genotek Inc.) and was quantified with Qubit™ dsDNA HS Assay Kit (Thermo Fisher Scientific Inc., Pittsburgh, PA, United States) (Utevsky et al., 2014). Five hundred ng of DNA was bisulfite-treated for cytosine to thymine conversion using the EZ DNA Methylation-Gold kit (Zymo Research, Irvine, CA, United States). DNA was then whole-genome amplified, fragmented, and hybridized to the HumanMethylationEPIC BeadChip (Illumina Inc., San Diego, CA, United States). The BeadChips were scanned using iSCAN (Illumina Inc.), and the methylation level (*β* value) was calculated for each queried CpG locus using the GenomeStudio Methylation Module software. As shown in Supplementary Figure S2, a quality check was conducted based on the Psychiatric Genomics Consortium-EWAS quality control pipeline (Ratanatharathorn et al., 2017) using CpGassoc (Barfield et al., 2012), an R package. Samples with probe detection call rates <90%, and those with an average intensity value of either <50% of the experiment-wide sample mean or <2,000 arbitrary units were excluded. Probes with detection *p* > 0.001, or those based on less than three beads, were set to missing, as were probes that cross-hybridized between autosomes and sex chromosomes. CpG sites with missing data for >10% of samples within cohorts were excluded from the analysis. After quality control, 852,775 probes were left for further analysis. Horvath’s multi-tissue clock mAge (Horvath, 2013) and the skin & blood clock mAge (Horvath et al., 2018) were calculated based on the online calculator (https://horvath.genetics.ucla.edu/html/dnamage/). The skin & blood clock is also known as a novel and highly robust DNAm age estimator for human fibroblasts, keratinocytes, buccal cells, endothelial cells, lymphoblastoid cells, skin, blood, and saliva samples, and has superior accuracy in blood and saliva samples than multi-tissue clock (Horvath et al., 2018). We regressed mAge on chronological age; the unstandardized residuals indicated epigenetic age acceleration. As saliva contains a heterogeneous mixture of cell types that differ in proportion in each sample, using the EpiDISH method (Teschendorff et al., 2017), we estimated the proportion of epithelial cells derived from salivary DNA (Epi) after the preprocessing processes similar to our previous study (Nishitani et al., 2021) and examined whether the proportion of epithelial cells affects mAge acceleration.

### MRI Acquisition and Voxel-Based Morphometry

Image acquisition of 30 participants was performed using a GE Discovery MR 750 3-T scanner (GE Healthcare, Milwaukee, WI). A T1-weighted anatomical dataset was obtained from each subject by a fast-spoiled gradient recalled imaging sequence (voxel size 1 × 1 × 1 mm, TE = 1.99 ms, TR = 6.38 ms, flip angle = 11°). Image acquisition of the 21 participants was carried out using a GE Signa PET/MR 3-T scanner (GE Healthcare, Milwaukee, WI). High-resolution structural whole-brain images were acquired using a 3D T1-weighted fast spoiled-gradient recalled imaging sequence (voxel size 1 × 1 × 1 mm, TE = 3.24 ms, TR = 8.46 ms, flip angle = 11°). Given that we used two scanners, a dummy-coded covariate (0 vs. 1) was added in the analyses. VBM data were analyzed using the Statistical Parametric Mapping software (SPM12; https://www.fil.ion.ucl.ac.uk/spm) implemented in MATLAB 2014a. The T1-weighted images were preprocessed using the VBM approach with modulation, where the images were first segmented into GM, white matter, cerebrospinal fluid, and skull/scalp compartments. Using the iterative high-dimensional normalization approach provided by Diffeomorphic Anatomical Registration through an Exponentiated Lie algebra algorithm (Ashburner, 2007), the segmented GM images were spatially normalized into the stereotaxic space of the Montreal Neurological Institute (MNI). The GM images had an isotropic voxel resolution of 1.5 mm^3^. Any volume change induced by normalization was adjusted via a modulation algorithm. The normalized modulated GM images were spatially smoothed by a Gaussian kernel of 8-mm full-width-at-half-maximum.

### Statistical Analysis

We conducted simple linear regression using heteroscedasticity-robust standard errors, with four types of reproductive effort indices as independent variables to predict mAge acceleration: parity status, number of deliveries, motherhood period, and cumulative motherhood period. We have employed this statistical technique for our analyses since it is no needed the assumption of homoscedasticity. To investigate the extent to which reproductive effort accounts for variance in mAge acceleration in the presence of various potential confounders (chronological age, PSI total, and Epi), multiple regression using robust standard errors was conducted. In the VBM analysis, chronological age, scanner (dummy coded as 0 and 1), and total brain volume were included as covariates of no interest in the design matrix to regress out their effects. The resulting set of voxel values used for each contrast generated a statistical parametric map of the *t*-statistic, SPM (*t*), which was transformed to a unit normal distribution [SPM (*Z*)]. The statistical threshold was set to *p* < 0.05, with family-wise error correction for multiple comparisons at the cluster level (height threshold of *Z* > 3.09). Significant clusters were localized in the Automated Anatomical Labelling atlases implemented in the MRIcron software package (https://www.nitrc.org/projects/mricron).

We conducted robust mediation analysis (Alfons et al., 2021) to assess whether the GM volume mediated the link between reproductive efforts and mAge acceleration. We included chronological age as the covariate in the model. The indirect effects of each model were tested by bootstrapping confidence intervals using the robmed package (Alfons et al., 2021). The model parameters were set to give bias-corrected 95% confidence intervals and to run 5,000 bootstrap resamples. All statistical analyses were performed with R 4.0.5 and SPM 12.

## Results

### Reproductive Effort and Epigenetic Age Acceleration

**FIGURE 1:**
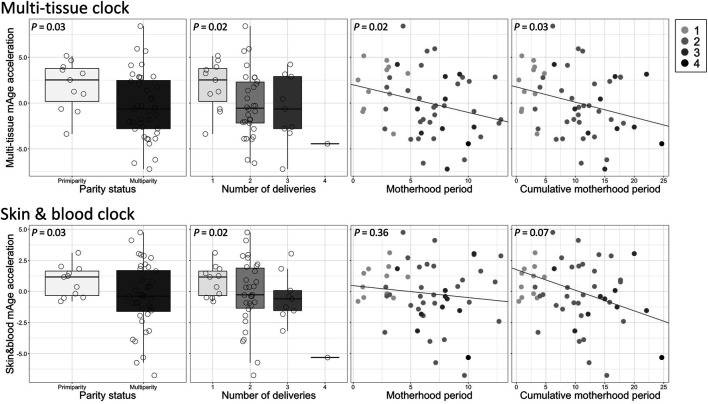
Associations between each reproductive effort and mAge acceleration. Top: multi-tissue clock; bottom: skin & blood clock. The gray shading in the legend on the upper right reflects the number of deliveries.

Among 353 and 391 CpG sites required to calculate Horvath’s multi-tissue clock mAge (Horvath, 2013) and skin & blood mAge (Horvath et al., 2018), we used 333 and 391 CpG probes, respectively. Seventeen probes were not included in the EPIC array to begin with and three probes (cg19167673, cg27413543, and cg14329157) were removed during data processing. As expected, mAge strongly correlated with chronological age (multi-tissue clock mAge: *R* ^2^ = 0.57, *t* = 8.13, *P* = 1.23e-10; skin & blood clock mAge: *R* ^2^ = 0.75, *t* = 12.17, *P* = 1.98e-16) (Supplementary Figure S3). The median absolute deviation from chronological age was 3.51 years for multi-tissue clock mAge, and 2.47 years for skin & blood clock mAge. These results were comparable with 2.7 years and 2.5 or 3.0 years, respectively, as reported by Horvath (Horvath, 2013); (Horvath et al., 2018). In simple linear regression analyses, mAge acceleration calculated by the multi-tissue clock was significantly negatively associated with parity status (*β* = −0.26, *t* = −2.30, *P* = 0.03), number of deliveries (*β* = −0.29, *t* = −2.45, *P* = 0.02), motherhood period (*β* = −0.28, *t* = −2.45, *P* = 0.02), and cumulative motherhood period (*β* = −0.29, *t* = −2.29, *P* = 0.03) (Table 2 and Figure 1). Conversely, mAge acceleration calculated by the skin and blood clock was significantly negatively associated with parity status (*β* = −0.21, *t* = −2.21, *P* = 0.03), and number of deliveries (*β* = −0.28, *t* = −2.44, *P* = 0.02), while no associations were found with other parameters (Table 2 and Figure 1). In other words, more reproductive efforts showed diminished mAge acceleration (Figure 1). PSI total (*β* = −0.42, *t* = −3.15, *P* = 0.003) and BDI-II (*β* = −0.29, *t* = −2.14, *P* = 0.04) were significantly associated with mAge acceleration calculated by the multi-tissue clock. No other demographic characteristics were associated with mAge acceleration for both calculations (Table 2). The correlation coefficients matrix between all the covariate combinations used in the analysis are shown in Supplementary Figure S4.

**TABLE 2:** Simple linear regression with robust standard errors for mAge accelerations.

	Multi-tissue clock			Skin & blood clock		
	*β*	*t*	*P*	*β*	*t*	*P*
Reproductive effort						
Parity status	−0.26	−2.30	0.03*	−0.21	−2.21	0.03*
Number of deliveries	−0.29	−2.45	0.02*	−0.28	−2.44	0.02*
Motherhood period	−0.28	−2.45	0.02*	−0.13	−0.92	0.36
Cumulative motherhood period	−0.29	−2.29	0.03*	−0.25	−1.84	0.07
Other variables						
Age	−0.09	−0.54	0.59	−0.04	−0.26	0.79
PSI total	−0.42	−3.15	0.003***	−0.14	−1.03	0.31
BDI-II	−0.29	−2.14	0.04*	−0.12	−0.98	0.33
Epi	−0.05	−0.35	0.73	−0.003	−0.02	0.98
Age at first childbirth	1.25	0.66	0.51	0.06	0.46	0.65
Age at last childbirth	−0.64	−0.36	0.72	0.02	0.18	0.86
Exclusive breastfeeding diet	0.09	0.59	0.56	0.24	1.93	0.06
Household income	0.04	0.27	0.79	0.18	1.44	0.16

*** *P < 0.05, **P < 0.01, P < 0.005.

BDI-II, The Beck Depression Inventory-II; mAge, DNA, methylation age; PSI, parenting stress index.

In addition to the simple linear regression, we also conducted multiple linear regression analyses to assess whether the other variables (chronological age, PSI total, and Epi) confounded the results (Table 3). Although chronological age and Epi were not significantly associated with mAge accelerations in the simple linear regression analysis, they were added in the multiple linear regression models as covariates. We chose PSI total instead of BDI-II as a covariate to avoid multi-collinearity issue in the regression model, as the two demonstrated a high correlation (*r* = 0.72, *P* = 2.85e-09); additionally, the association between PSI total and mAge acceleration was greater than that of BDI-II (Table 2). Results showed that all four reproductive effort indices were significantly negatively associated with mAge acceleration calculated by the multi-tissue clock, even after adjusting for the confounding effects (*Ps* < 0.05, Table 3). Although the results of mAge acceleration calculated by the skin & blood clock showed significant associations with parity status (*P* = 0.03), number of deliveries (*P* = 0.02), and cumulative motherhood period (*P* = 0.04) in the multiple linear regression model, these models were not significant (Table 3). Hence, we used mAge acceleration based on the multi-tissue clock for subsequent analyses.

**TABLE 3:** Multiple linear regression with robust standard errors for mAge accelerations.

		Multi-tissue clock				Skin & blood clock			
		Parity status	Number of deliveries	Motherhood period	Cumulative motherhood period	Parity status	Number of deliveries	Motherhood period	Cumulative motherhood period
Reproductive effort	*β*	−0.25	−0.26	−0.29	−0.28	−0.21	−0.27	−0.14	−0.27
	*t*	−2.26	−2.52	−2.15	−2.14	−2.23	−2.36	−0.91	−2.09
	*P*	0.03*	0.02*	0.04*	0.04*	0.03*	0.02*	0.37	0.04*
Age	*β*	0.07	0.02	0.14	0.10	0.05	0.01	0.06	0.09
	*t*	0.43	0.15	0.74	0.61	0.34	0.08	0.38	0.635
	*P*	0.67	0.88	0.46	0.54	0.73	0.94	0.70	0.53
PSI total	*β*	−0.42	−0.41	−0.41	−0.42	−0.14	−0.13	−0.14	−0.14
	*t*	−3.06	−3.23	−3.05	−0.32	−0.96	−0.90	−0.97	−0.96
	*P*	0.004***	0.002***	0.004***	0.003***	0.34	0.37	0.34	0.34
Epi	*β*	0.03	0.03	0.06	0.06	0.04	0.03	0.04	0.06
	*t*	0.24	0.20	0.42	0.41	0.24	0.22	0.27	0.39
	*P*	0.81	0.85	0.68	0.69	0.81	.83	0.79	0.70
Model	*R* ^*2*^	0.16	0.18	0.17	0.17	−0.02	0.015	−0.05	0.004
	*F*	4.99	4.84	3.66	3.98	1.98	1.86	0.46	1.48
	*P*	0.002***	0.002***	0.01*	0.007**	0.11	0.13	0.77	0.22

*** *P < 0.05, **P < 0.01, P < 0.005.

### VBM and Path Analysis

**FIGURE 2:**
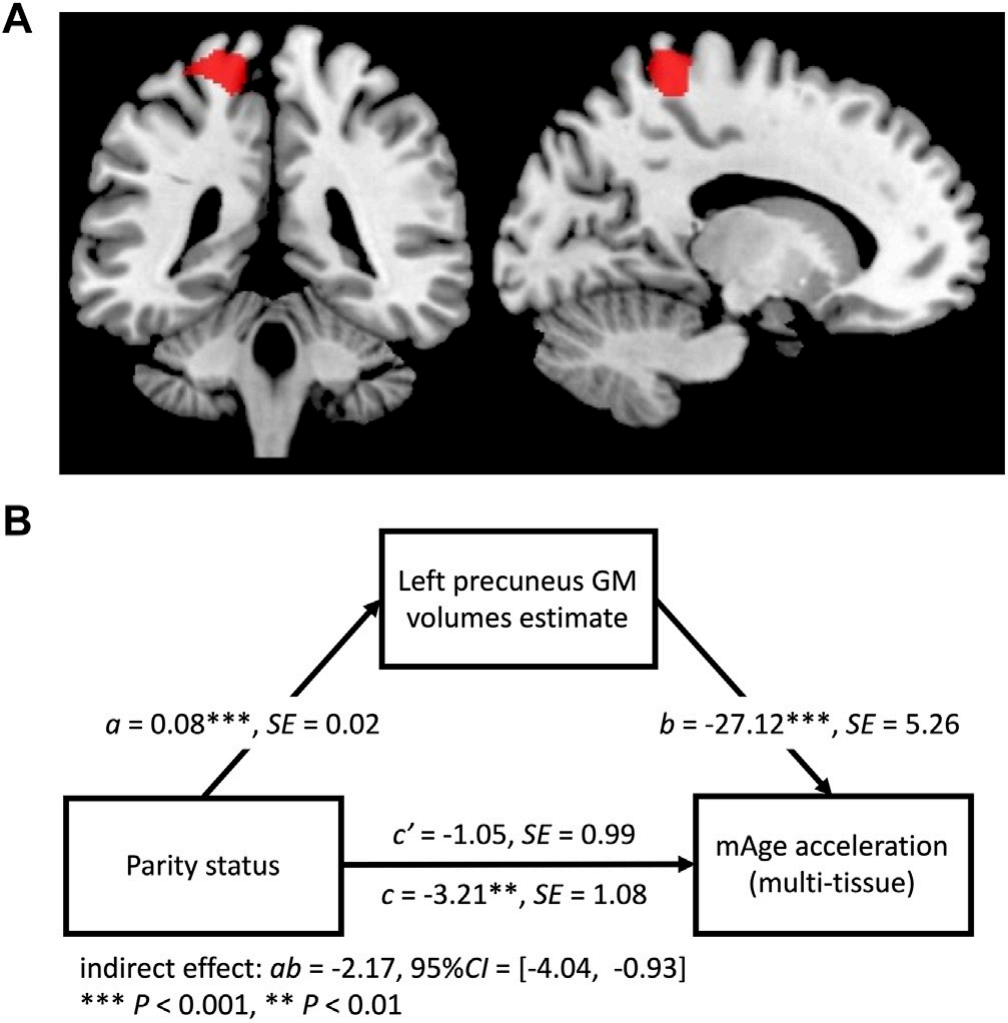
**(A)** Regions of gray matter (GM) volume negatively associated with mAge acceleration (red; threshold *p* < 0.05, family-wise error corrected cluster level). **(B)** Path model for the mediation effect. GM volume of the left precuneus was a mediator in the relationship between parity status and mAge acceleration.

The VBM results showed that mAge acceleration was negatively correlated with GM volume within a cluster in the left precuneus (Montreal Neurological Institute [MNI] coordinates: *x* = −17, *y* = −39, *z* = 68; cluster size = 727 voxels; *P* = 0.04, family-wise error [FWE] corrected cluster level; Figure 2A). There was a significant indirect effect of the precuneus GM volume on parity status and mAge acceleration (indirect effect = −2.17, 95% Confidence Interval = [−4.04, −0.93]; Figure 2B). No other reproductive efforts had a significant indirect effect.

## Discussion

This study examined the relationship between reproductive effort, mAge acceleration, and brain structure in mothers of children in early childhood. Our results showed that their reproductive effort—regarding parity status, number of deliveries, motherhood period, and cumulative motherhood period—was associated with mAge deceleration, even after adjusting for potential confounders. Although previous studies have examined the relationship between mAge acceleration or telomere length and the number of deliveries as a representative variable, this is the first time that mAge acceleration has been simultaneously examined in relation to the other reproductive effort indices, also considering daily parenting stress. We also found that the precuneus GM volume increased as mAge deceleration occurred. We also observed a mediation effect of greater left precuneus GM volume on the relationship between parity status and mAge deceleration. This suggests that the precuneus—a central node in the human brain that supports complex cognition and behavior—may be associated with age deceleration in child-rearing mothers. This also suggests that multiparity between two to four births lead to maternal brain changes in the left precuneus, which contributes more to mAge deceleration than just one birth.

Our results were limited as we only included mothers who gave birth to one to four children; thus, we do not know how this would have affected women with more than four births. Still, among mothers who gave birth to one to four children, our results seemed to contradict the LHT (Hill and Kaplan, 1999). Our results suggest that mothers who gave birth to less than four children and have currently been rearing them have aged more slowly, depending on their reproductive efforts. Our findings are consistent with previous large demographic studies reporting that deliveries of less than four children extended, rather than diminished, life expectancy (Dior et al., 2013; Bolund et al., 2016; Shadyab et al., 2017). We speculate that this avoids a downward trend in the birth rate, since having only one offspring born to each couple would lead to a decline in population in a monogamous society. While childbearing certainly comes at the cost of energy based on the LHT, giving birth to two or more children while having fewer than four may benefit the mother’s lifespan, even accounting for the tradeoffs of childbirth.

One possible biological cause underlying this phenomenon could be due to maternal brain alteration. Human mothers’ brains undergo dynamic structural and functional changes during pregnancy and the early postpartum period to facilitate their psychological and behavioral adaptation to parenting (Kim, 2016; Hoekzema et al., 2017; Zhang et al., 2019; Luders et al., 2020). This transition to the maternal brain seems to particularly enhance the functioning of the reward, social information, and emotion regulation circuits (Ho et al., 2014). We identified that mAge deceleration associated with parity status was linked to an increase in precuneus GM volume in mothers, which is similar to the findings of the study by Luders et al. where an increase in precuneus GM volume was observed at 4–6 weeks postpartum, compared with 1–2 days postpartum (Luders et al., 2020). The precuneus is included in the social information circuit and is a hub for the default mode network (DMN), which involves empathy, as well as self-monitoring and reflection (Utevsky et al., 2014). In other words, becoming a mother of two or more children may have led to more complex social interactions, which may have contributed to a greater precuneus GM volume than in the past. It has also been found that patients with Alzheimer’s disease (AD) and in the prodromal stage of AD have decreased DMN functions and precuneus GM volume compared to controls (Goto et al., 2015). Using postmortem brains, a telomere length analysis of each brain region comprising the DMN showed that the precuneus telomere length was shorter in AD and the prodromal stage of AD than in controls, whereas there were no differences in the frontal, inferior temporal, posterior cingulate gyrus or visual cortex (Mahady et al., 2020). Furthermore, this telomere length reduction in the precuneus also correlated with cognitive task performance. One of the main symptoms of PTSD is an elevated rumination characterized by repetitive, negative self-focused cognition; it has been reported that reduced functional connectivity between the isthmus cingulate and the left precuneus within the DMN is linked to this high level of rumination (Philippi et al., 2020). Lifetime trauma burden and suffering from both current and lifetime PTSD have been reported to cause GrimAge acceleration (Katrinli et al., 2020). These studies support the notion that aging and mental stress that accelerates aging may affect the functional connectivity between the precuneus and the other brain network region. The sophistication of the social information and DMN functions involved in becoming a mother may have contributed to the mAge deceleration via enhancing precuneus function and volume; however, this finding is not related to the number of deliveries—which accounts for the major reproductive effort—but rather to parity status (whether a baby is born to a mother once or twice or more), and is not a model that is valid as the number of deliveries increases.

Our results were partially inconsistent with previous studies. Ryan et al. reported mAge acceleration in mothers who had given birth to one to five children, as well as in nulliparous women (Ryan et al., 2018). However, their study participants were 20–22 years-old, which is relatively young compared with the average age of a primipara in modern, developed countries. Some reports suggest that a higher age at first birth is associated with longer telomere length (Fagan et al., 2017) and longer life expectancy (Shadyab et al., 2017). Thus, the association may be linear, rather than U-shaped, when the age at first birth is relatively young, as in this previous study. In addition, various environmental factors have been reported to influence mAge acceleration, most notably stress (Hoare et al., 2020; Katrinli et al., 2020). While Ryan et al. adjusted for socioeconomic status, they did not consider the effects of psychological aspects, such as parental stress; therefore, an analysis adjusting for these effects may also be necessary. In our results, higher parental stress, not only reproductive effort, was associated with the deceleration of aging. Conversely, Kresovich et al. reported accelerated aging in mothers who gave birth to one to four or more children, as well as in nulliparous women (Kresovich et al., 2019). However, their study was retrospective and included those who were currently out of the child-rearing phase (an average age of 55 years when the blood samples were collected), which is different from the present study, involving mothers of children in early childhood. Their results may thus be confounded by menopause (Gray et al., 2014) and the higher genetic risks for developing breast cancer, which is a unique characteristic of this cohort population (Sandler et al., 2017). By contrast, Barha et al. found longer telomeres in mothers who gave birth to one to six babies (Barha et al., 2016). Although this study did not measure mAge acceleration, telomere length results indicate that age deceleration may have occurred, perhaps akin to our findings. Additionally, our studies were similar in that the DNA was saliva-derived. Our method of estimating mAge by using Horvath’s multi-tissue clock should not be noticeably affected, even if the tissue from which the DNA was derived was different (Horvath, 2013). Still, the similarities regarding the type of sample may not be particularly relevant. Additionally, the participants in their study were, on average, 39.4 years old, premenopausal, and probably still rearing their children. This consistency in age and rearing young children may be related to the directional consistency of the two results. Although their results were linearly regressed, on closer inspection, an inverted U-shaped approximation with a peak at two to three births appears to be more appropriate.

There are at least six potential limitations concerning our study’s results. First, the small number of participants; we only had one participant with more than four children, limiting the ability to observe the U-shaped association that we hypothesized. Small group sizes may preclude drawing significant conclusions and limit generalizability of the results; thus, we will confirm these pilot results by increasing the sample size in future studies. Second, there were no nulliparous participants. Shadyab et al. (2017) reported that mothers had a longer life expectancy than nulliparous women (Shadyab et al., 2017). It is also known that changes in brain morphology and cognitive function occur when women become mothers (Hoekzema et al., 2017; Zhang et al., 2019; Luders et al., 2020); therefore, it is necessary to include nulliparous women to show the association between mAge acceleration and precuneus GM volume with brain materialization. Third, we used saliva DNA samples for mAge and mAge acceleration calculation. This limits the use of other newly developed blood DNA composite biomarkers, including: PhenoAge, which more effectively captures the epigenetic biomarkers of physiological age and discriminates between morbidity and mortality more definitively in individuals of the same chronological age (Levine et al., 2018); and GrimAge—named after the Grim Reaper—which predicts lifespan and healthspan in units of years (Lu et al., 2019). However, Horvath’s multi-tissue clock can be used for any type of tissues, enabling a direct comparison with most previous studies based on blood DNA (Ryan et al., 2018; Kresovich et al., 2019). Forth, there was a significant association that higher the PSI, the more age deceleration based on the multi-tissue clock (*p* = 0.003), but not for skin & blood clock (*p* = 0.31). Zannas et al. (2015) have reported that lifetime stress accelerates epigenetic aging since the multi-tissue clock has 85 probes located within glucocorticoid response elements (Zannas et al., 2015). Thus, these specific probes included in the multi-tissue clock might have affected the association with PSI. However, the effect directionality of our results was different. This may suggest that the age deceleration was not effortlessly obtained as it was also associated with the length of the motherhood period. Fifth, we do not have information whether these participants cohabit with their parent or parent-in-law. According to the public demographics, nuclear families in Japan were 82.7% (2016) (Graphical Review of Japanese Household, 2016) and this might have influenced the results. Finally, we have focused only on mAge acceleration, but it is necessary to explore the relationship with the genome-wide profile, which is a future research goal.

## Conclusion

Despite these limitations, our results suggest that reproductive effort in mothers may refine the brain structures involved in the sociability required for parenting, conversely influencing mAge acceleration and longevity. Our results also suggest that the mAge deceleration associated with changes in the precuneus may be one of the phenomena linked to the maternalization of the brain.

## Data Availability

The original contributions presented in the study are included in the article/Supplementary Material, further inquiries can be directed to the corresponding authors.
