# Supplementary material for: Epigenetic Clock Deceleration and Maternal Reproductive Efforts: Associations With Increasing Gray Matter Volume of the Precuneus
Source: Front Genet. 2022 Mar 1;13:803584. doi: 10.3389/fgene.2022.803584 (PMC8926035; doi:10.3389/fgene.2022.803584)
Supplement: Supplementary file 1 [file DataSheet2.docx]

**Supplementary Figure S1. Detailed graphical plot indicating individual reproductive effort**

(A) Plot demonstrating when participating mothers gave birth and how many children they had been rearing until the day of the experiment, E. The color scale indicates the number of deliveries. (B) Age (years) of each child from 1^st^ to 4^th^ child, and total age (years) of all children (cumulative motherhood period).

**Supplementary Table S1. Demographic characteristics between the number of deliveries groups**

| Number of deliveries | 1 | 2 | 3 | 4 |
| --- | --- | --- | --- | --- |
| *n* | 11 | 30 | 9 | 1 |
| Age (years), *Mean* (*SD*) | 33.1 (4.2) | 36.6 (4.7) | 34.0 (2.4) | 35.1 (NA) |
| PSI (Total) | 184.2 (23.4) | 194.0 (45.8) | 189.4 (37.6) | 218.0 (NA) |
| Epi (%) | 14.6 (5.8) | 16.3 (6.1) | 16.2 (5.1) | 13.7 (NA) |
| Household Income (currency = JPY), *n* (%)  Less than 3 million  3–5 million  5–10 million  More than 10 million | 0 (0)  6 (54.5)  5 (45.5)  0 (0) | 2 (6.7)  13 (43.3)  13 (43.3)  2 (6.7) | 1 (11.1)  5 (55.6)  3 (33.3)  0 (0) | 0 (0)  1 (100)  0 (0)  0 (0) |
| Total brain volume (mm^3^) | 1083.8 (68.2) | 1081.1 (58.8) | 1106.3 (92.5) | 1185.5 (NA) |

PSI, Parenting Stress Index (Abidin, 1995; Namara et al., 1999); Epi, proportion of buccal epithelial cells

**Supplementary Figure S2. A flowchart of preprocessing procedures for mAge calculation**

**Supplementary Figure S3. Linear regression of methylation age (mAge) prediction using Horvath’s (A) multitissue clock and (B) skin & blood clock**

The individual unstandardized residuals (mAge acceleration) are indicated by the size of the points and their distance from the regression line. The gray scale indicates the number of deliveries.

**Supplementary Figure S4. Pearson correlation analysis matrix for all covariate combinations**

The size and color of the circles reflect the correlation coefficients for each combination in the correlation analysis.
